# Final results of the global and Asia cohorts of KAMILLA, a phase IIIB safety trial of trastuzumab emtansine in patients with HER2-positive advanced breast cancer

**DOI:** 10.1016/j.esmoop.2022.100561

**Published:** 2022-09-07

**Authors:** R. Wuerstlein, P. Ellis, F. Montemurro, A. Antón Torres, S. Delaloge, Q. Zhang, X. Wang, S. Wang, Z. Shao, H. Li, A. Rachman, M. Vongsaisuwon, H. Liu, S. Fear, C. Peña-Murillo, C. Barrios

**Affiliations:** 1 University Hospital Munich, Department of Obstetrics and Gynecology, Breast Center and CCC Munich, LMU, Munich, Germany; 2 Guy’s Hospital and Sarah Cannon Research Institute, London, UK; 3 Candiolo Cancer Institute, FPO-IRCCS, Candiolo, Italy; 4 Miguel Servet University Hospital and Aragon Health Research Institute (IISA), Zaragoza, Spain; 5 Institut Gustave Roussy, Villejuif, France; 6 Harbin Medical University Cancer Hospital, Nangang, Harbin; 7 Zheijang Cancer Hospital, Gonghshu District, Hangzhou; 8 Sun Yet-sen University Cancer Center, Yuexiu District, Guangzhou; 9 Fudan University Shanghai Cancer Center, Xuhui District, Shanghai; 10 Key Laboratory of Carcinogenesis and Translational Research (Ministry of Education/Beijing), Department of Breast Oncology, Peking University Cancer Hospital & Institute, Hai-Dian District, Beijing, China; 11 MRCCC Siloam Semanggi Hospital, Daerah Khusus Ibukota, Jakarta, Indonesia; 12 King Chulalongkorn Memorial Hospital, Pathum Wan, Bangkok, Thailand; 13 F. Hoffmann-La Roche, Basel, Switzerland; 14 Oncology Research Center HSL, PUCRS, Latin American Cooperative Oncology Group, Porto Alegre, Brazil

**Keywords:** trastuzumab emtansine (TDM-1), HER2-positive metastatic breast cancer, KAMILLA, safety, Asia

## Abstract

**Background:**

KAMILLA is a single-arm safety study of trastuzumab emtansine (T-DM1) in patients with human epidermal growth factor receptor 2 (HER2)-positive advanced breast cancer (BC; NCT01702571). We report the final analysis of cohort 2 (Asia) within the context of published cohort 1 (Global) findings.

**Methods:**

Patients had HER2-positive, locally advanced, or metastatic BC progressing after chemotherapy and anti-HER2 therapy or ≤6 months after adjuvant therapy. The primary objective was to further evaluate T-DM1 (3.6 mg/kg, administered intravenously every 3 weeks) safety/tolerability, including the following adverse events of primary interest (AEPIs): grade ≥3 AEPIs (hepatic events, allergic reactions, thrombocytopenia, hemorrhage events), all grade ≥3 treatment-related AEs, and all-grade pneumonitis.

**Results:**

KAMILLA enrolled 2185 patients (cohort 1, *n* = 2003; cohort 2, *n* = 182) as of 31 July 2019. Of these, 2002 and 181 per cohort were treated and included in the safety population. Approximately 70% of patients had two or more previous treatment lines in the metastatic setting. Median T-DM1 exposure was 5.6 and 5.0 months per cohort; median follow-up was 20.6 and 15.1 months. The overall AEPI rate was higher in cohort 2 (93/181; 51.4%) versus cohort 1 (462/2002; 23.1%), mostly driven by a higher grade ≥3 thrombocytopenia rate in cohort 2. In cohort 2, grade ≥3 thrombocytopenia was not associated with grade ≥3 hemorrhagic events and most (128/138) fully resolved. Grade ≥3 treatment-related AEPI rates were 18.4% (cohort 1) and 48.6% (cohort 2), the latter mainly due to thrombocytopenia. Any-grade pneumonitis rates were 1.0% and 2.2%. No new safety signals were identified. Median (95% confidence interval) progression-free survival was 6.8 months (5.8-7.6 months) and 5.7 months (5.5-7.0 months) in cohorts 1 and 2, respectively; median overall survival was 27.2 months (25.5-28.7 months) and 29.5 months (21.1 months to non-estimable). In both cohorts, median progression-free survival and overall survival decreased with increasing prior therapy lines.

**Conclusions:**

Cohort 2 results aligned with previous findings in Asian patients, supporting the manageable safety profile and use of T-DM1 in advanced BC.

## Highlights

• KAMILLA safety results for cohorts 1 (global; *n* = 2002) and 2 (Asia; *n* = 181) aligned with results from prior T-DM1 mBC trials.
• The overall rate of adverse events of primary interest (AEPIs) was higher in cohort 2 (51.4%) versus cohort 1 (23.1%).
• The higher AEPI rate was mostly due to a higher grade ≥3 thrombocytopenia event rate in cohort 2, most of which resolved.
• Median PFS and OS were similar for both cohorts, and decreased with increasing prior therapy lines.
• The manageable safety profile and efficacy of T-DM1 further support its favorable benefit/risk balance.

## Introduction

Trastuzumab emtansine (T-DM1) is approved worldwide for the treatment of patients with human epidermal growth factor receptor 2 (HER2)-positive, metastatic breast cancer (mBC) who previously received trastuzumab and a taxane, separately or in combination.^1^^,^^2^ The first antibody-drug conjugate approved for breast cancer (BC) treatment, T-DM1 is composed of the HER2-targeted antibody, trastuzumab, stably linked to DM1, a cytotoxic microtubule-inhibiting drug.^3^ This design enables delivery of DM1 specifically to HER2-positive cells, thereby maximizing the therapeutic potential of DM1 while limiting off-target effects. The approval of T-DM1 in the mBC setting in the USA and Europe (both 2013), as well as in China (2021) and other Asian countries, was based on results from the phase III EMILIA trial of patients with HER2-positive advanced BC who received prior treatment with trastuzumab and a taxane.^4^ In EMILIA, patients who received T-DM1 versus lapatinib plus capecitabine had significantly longer progression-free survival (PFS; median 9.6 months versus 6.4 months; *P* < 0.001) and overall survival (OS; median 30.9 months versus 25.1 months; *P* < 0.001), and experienced fewer grade ≥3 adverse events (AEs; 41% versus 57%).^4^ Additionally, in the phase III TH3RESA study, patients with prior progression on two or more anti-HER2 regimens experienced significantly improved PFS and OS with T-DM1 versus treatment of physician’s choice (median PFS, 6.2 versus 3.3 months; *P* < 0.0001; median OS, 22.7 versus 15.8 months; *P* = 0.0007), and had fewer grade ≥3 AEs (32% versus 43%).^5^^,^^6^ Real-world analyses of T-DM1 have generally aligned with results from these clinical trials, providing further evidence for use of this agent in previously treated mBC.^7^, ^8^, ^9^, ^10^

KAMILLA (NCT01702571) is a multicenter, phase IIIb trial of T-DM1 in patients with HER2-positive mBC or locally advanced BC, and was conducted as a post-approval safety measure to fulfill a commitment to the European Medicines Agency (EMA). KAMILLA comprises two cohorts: a larger global cohort 1 (*n* = 2002 treated patients), and a smaller Asia cohort 2 (*n* = 181 treated patients). In the primary analysis of cohort 1, safety results were consistent with those from prior randomized studies,^4^^,^^5^ with AEs and grade ≥3 AEs occurring in 93.0% and 37.5% of patients, respectively.^11^ Median PFS and median OS were 6.9 and 27.2 months, respectively, and both median PFS and OS decreased numerically with increasing treatment lines.

Previous studies have shown differences in AE rates between patients of Asian and non-Asian descent, such as a higher incidence of grade ≥3 thrombocytopenia in Asian patients.^12^ To further understand the safety profile of T-DM1, this final analysis assessed the safety of T-DM1 in cohort 2 from KAMILLA, in the context of previous findings from the cohort 1 analysis.

## Materials and methods

### Study design

KAMILLA (NCT01702571) is an international, multicenter, open-label, single-arm, phase IIIb study comprising two cohorts. Cohort 1 included 2003 patients from 40 countries, and cohort 2 included 182 patients from Asia.^11^ Of these, 2002 patients from cohort 1 and 181 patients from cohort 2 (China, *n* = 154; Thailand, *n* = 15; Indonesia, *n* = 12) were included in the treated (safety) population. All treated patients received T-DM1 (3.6 mg/kg every 3 weeks intravenously) until progressive disease, unacceptable toxicity, patient withdrawal, or death. AE assessment occurred on an ongoing basis, and survival follow-up occurred every 6 months (±14 working days) until death, withdrawal of consent, or loss to follow-up.

### Patients

Eligible patients were males or females ≥18 years of age with HER2-positive mBC or locally advanced BC. Patients had one or more prior treatment in the early BC or mBC setting, received a prior anti-HER2 agent and chemotherapy, and had progressed on metastatic treatment or within 6 months of completing adjuvant therapy. Other key inclusion criteria included measurable and/or non-measurable disease, Eastern Cooperative Oncology Group (ECOG) performance status of 0-2, left ventricular ejection fraction ≥50%, and adequate organ function. Key exclusion criteria included prior T-DM1 treatment, grade ≥3 peripheral neuropathy, and symptomatic central nervous system (CNS) metastases or CNS-limited metastatic disease.

All patients provided written informed consent. The study was conducted in accordance with the International Conference on Harmonisation of Technical Requirements for Registration of Pharmaceuticals for Human Use–Good Clinical Practice: Consolidated Guideline, and was approved by the institutional review board at each site.

### Outcomes and statistical analysis

The primary objective was to further evaluate the safety and tolerability of T-DM1. The safety population included all patients who received ≥1 dose of study medication. AEs were categorized using Medical Dictionary for Regulatory Activities version 22.1, and were graded per National Cancer Institute Common Terminology Criteria for Adverse Events version 4.0.^13^ Grade ≥3 AEs of primary interest (AEPIs; specifically, hepatic events, allergic reactions, thrombocytopenia, and hemorrhage events), all grade ≥3 treatment-related AEs, and all-grade pneumonitis were evaluated. Safety data were summarized descriptively, with no formal statistical tests carried out.

Secondary endpoints included PFS and OS in the intent-to-treat population, which consisted of all enrolled patients. Time-to-event endpoints were evaluated per standard statistical methodology, and were also evaluated in subgroups by prior treatment lines.

## Results

### Patients

**Figure 1:**
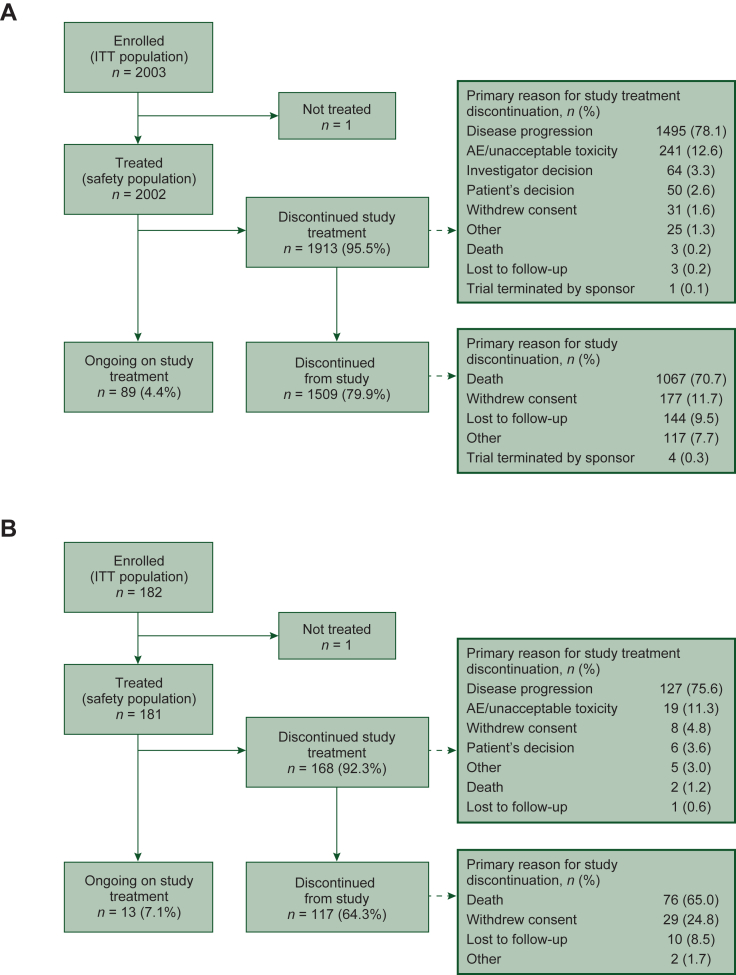
Patient disposition. (A) Cohort 1—global^a^; (B) cohort 2—Asia. AE, adverse event; ITT, intent-to-treat. ^a^Patient disposition diagram for cohort 1 previously published in Montemurro et al., *Eur J Cancer.* 2019;109:92–102.^11^

A total of 2003 patients were enrolled in cohort 1 between November 12, 2012, and September 29, 2014, and 182 patients were enrolled in cohort 2 between 14 December 2014 and 17 May 2017. By database lock (31 January 2017 for cohort 1 and 31 July 2019 for cohort 2), 2002 and 181 patients in each cohort, respectively, received one or more study dose and were included in the safety population (Figure 1). Median follow-up duration was 20.6 months (range 0-89 months) for cohort 1 and 15.1 months (range 1-57 months) for cohort 2 as of the respective database locks. Baseline characteristics were generally similar between the two cohorts (Table 1), with some differences. A greater percentage of patients in cohort 2 were younger and had hormone receptor-negative disease than cohort 1 patients, and there were differences in ECOG performance status at screening. Furthermore, whereas all patients in cohort 2 previously received neoadjuvant or adjuvant chemotherapy, only 73% of patients in cohort 1 received these therapies. Approximately 70% of patients in each cohort had two or more previous treatment lines in the metastatic setting. Deaths occurred in 53.5% of patients in cohort 1 and 42.5% of patients in cohort 2, primarily due to disease progression (51.3% and 40.3% for each cohort, respectively); deaths due to AEs occurred in 2.2% of patients in cohort 1 and 1.7% of patients in cohort 2.

**Table 1:** Patient demographics and baseline characteristics

	Cohort 1^a^*n* = 2002	Cohort 2 *n* = 181	Total *N* = 2183
Sex			
Female	1988 (99.3)	181 (100.0)	2169 (99.4)
Male	14 (0.7)	0 (0.0)	14 (0.6)
Age, years			
Mean (SD)	54.5 (11.36)	49.0 (10.07)	54.1 (11.35)
Median (min, max)	55.0 (26, 88)	51.0 (25, 67)	54.0 (25, 88)
Age group			
<65 years	1629 (81.4)	172 (95.0)	1801 (82.5)
≥65 years	373 (18.6)	9 (5.0)	382 (17.5)
≥75 years	101 (5.0)	0 (0.0)	101 (4.6)
ECOG performance status at screening			
0	1110 (55.4)	76 (42.0)	1186 (54.3)
1	775 (38.7)	101 (55.8)	876 (40.1)
2	115 (5.7)	4 (2.2)	119 (5.5)
Missing	2 (0.1)	0 (0.0)	2 (0.1)
Time from diagnosis to first metastases, years			
N	1993	180	2173
Mean (SD)	3.0 (3.72)	2.2 (2.13)	3.0 (3.63)
Median (min, max)	2.0 (0, 24)	1.7 (0, 13)	1.9 (0, 24)
ER/PR responsiveness at diagnosis			
ER- and/or PR-positive	1232 (61.5)	77 (42.5)	1309 (59.9)
ER- and PR-negative	733 (36.6)	102 (56.4)	835 (38.3)
Prior number of lines of metastatic treatment			
0 or 1	594 (29.7)	62 (34.3)	656 (30.1)
2	446 (22.3)	50 (27.6)	496 (22.7)
3	358 (17.9)	28 (15.5)	386 (17.7)
4+	517 (25.8)	33 (18.2)	550 (25.2)
Missing	87 (4.3)	8 (4.4)	95 (4.4)
Type of systemic cancer therapy before study			
No. of patients with ≥1 chemotherapy	1998 (99.8)	181 (100.0)	2179 (99.8)
Neoadjuvant	499 (24.9)	43 (23.8)	542 (24.8)
Adjuvant	960 (48.0)	129 (71.3)	1089 (49.9)
Antiestrogen (hormonal) therapy	1173 (58.6)	74 (40.9)	1247 (57.1)
Visceral disease at screening	1561 (78.0)	157 (86.7)	1718 (78.7)
CNS metastases at baseline (brain)	398 (19.9)	44 (24.3)	442 (20.2)
Other metastases at baseline			
Bone	1091 (54.5)	85 (47.0)	1176 (53.9)
Lymph nodes	918 (45.9)	100 (55.2)	1018 (46.6)
Lung	873 (43.6)	102 (56.4)	975 (44.7)
Hepatic	817 (40.8)	92 (50.8)	909 (41.6)
Chest wall	414 (20.7)	42 (23.2)	456 (20.9)
Brain	398 (19.9)	44 (24.3)	442 (20.2)
Skin	254 (12.7)	8 (4.4)	262 (12.0)
Pleural effusion	222 (11.1)	12 (6.6)	234 (10.7)
Other abdominal	176 (8.8)	11 (6.1)	187 (8.6)
Opposite breast	109 (5.4)	23 (12.7)	132 (6.0)
Ascites	43 (2.1)	3 (1.7)	46 (2.1)
Other	34 (1.7)	6 (3.3)	40 (1.8)
Pericardium	18 (0.9)	3 (1.7)	21 (1.0)
Platelet count (10^9^/l) at baseline			
No. of patients	1948	180	2128
Mean (SD)	270.7 (81.31)	246.4 (74.89)	268.6 (81.05)
Median (min, max)	258.0 (14, 757)	244.5 (101, 496)	257.0 (14, 757)

All values are *n* (%) unless otherwise noted.

CNS, central nervous system; ECOG, Eastern Cooperative Oncology Group; ER, estrogen receptor; PR, progesterone receptor; SD, standard deviation.

a One patient in cohort 1 had prior pertuzumab exposure.

### Safety

Median duration of T-DM1 exposure was 5.6 months (range 0-46 months) for cohort 1 and 5.0 months (range 0-31 months) for cohort 2. The percentage of patients with a dose reduction at any cycle was 22.5% for cohort 1 and 60.2% for cohort 2. Among the 1913 patients in cohort 1 and 168 patients in cohort 2 who discontinued study treatment, the most common reason for discontinuation in both cohorts was disease progression (78.1% and 75.6%, respectively). Of 237 (11.8%) of patients in cohort 1 and 20 patients (11.0%) in cohort 2 with details available for AEs leading to treatment discontinuation, decreased platelet count/thrombocytopenia (cohort 1, 2.2%; cohort 2, 2.8%) and increased blood bilirubin (cohort 1, 1.2%; cohort 2, 1.7%) occurred in >0.5% of patients in each cohort.

Incidence of AEPIs (grade ≥3 thrombocytopenia, hepatic events, allergic reactions, hemorrhage; all-grade pneumonitis; grade ≥3 AEs related to T-DM1) was higher in cohort 2 [51.4%; 95% confidence interval (CI) 43.9% to 58.9%] than in cohort 1 (23.1%; 95% CI 21.2% to 25.0%), mostly driven by a higher grade ≥3 thrombocytopenia rate in cohort 2 versus cohort 1 (36.5% versus 3.7%; Table 2). The all-grade pneumonitis rate was 1.0% in cohort 1 and 2.2% in cohort 2, and the rate of grade ≥3 treatment-related AEs was 18.4% and 48.6%, respectively.

**Table 2:** AEPIs

System organ class preferred term	Cohort 1 (*n* = 2002)		Cohort 2 (*n* = 181)		Total (*N* = 2183)	
	*n* (%)	95% CI	*n* (%)	95% CI	*n* (%)	95% CI
No. of patients with ≥1 qualifying event	462 (23.1)	21.2-25.0	93 (51.4)	43.9-58.9	555 (25.4)	23.6-27.3
Grade ≥3 thrombocytopenia						
No. of patients with ≥1 event	74 (3.7)	2.9-4.6	66 (36.5)	29.5-43.9	140 (6.4)	5.4-7.5
Grade 3	62 (3.1)		58 (32.0)		120 (5.5)	
Grade 4	12 (0.6)		23 (12.7)		35 (1.6)	
Grade 5	0 (0.0)		0 (0.0)		0 (0.0)	
Grade ≥3 hepatic events						
No. of patients with ≥1 event	139 (6.9)	5.9-8.1	22 (12.2)	7.8-17.8	161 (7.4)	6.3-8.6
Grade 3	132 (6.6)		22 (12.2)		154 (7.1)	
Grade 4	11 (0.5)		1 (0.6)		12 (0.5)	
Grade 5	6 (0.3)		0 (0.0)		6 (0.3)	
Grade ≥3 allergic reactions						
No. of patients with ≥1 event	46 (2.3)	1.7-3.1	2 (1.1)	0.1-3.9	48 (2.2)	1.6-2.9
Grade 3	40 (2.0)		2 (1.1)		42 (1.9)	
Grade 4	5 (0.2)		0 (0.0)		5 (0.2)	
Grade 5	1 (0.0)		0 (0.0)		1 (0.0)	
Grade ≥3 hemorrhage						
No. of patients with ≥1 event	46 (2.3)	1.7-3.1	3 (1.7)	0.3-4.8	49 (2.2)	1.7-3.0
Grade 3	40 (2.0)		2 (1.1)		42 (1.9)	
Grade 4	6 (0.3)		1 (0.6)		7 (0.3)	
Grade 5	2 (0.1)		0		2 (0.1)	
All-grade pneumonitis						
No. of patients with ≥1 event^a^	21 (1.0)	0.7-1.6	4 (2.2)	0.6-5.6	25 (1.1)	0.7-1.7
Grade 3	3 (0.1)		0 (0.0)		3 (0.1)	
Grade 4	2 (0.1)		0 (0.0)		2 (0.1)	
Grade 5	4 (0.2)		0 (0.0)		4 (0.2)	
Grade ≥3 AEs related to T-DM1						
No. of patients with ≥1 event	368 (18.4)	16.7-20.1	88 (48.6)	41.1-56.1	456 (20.9)	19.2-22.7
Grade 3	344 (17.2)		78 (43.1)		422 (19.3)	
Grade 4	31 (1.5)		27 (14.9)		58 (2.7)	
Grade 5	12 (0.6)		1 (0.6)		13 (0.6)	

AEPI, adverse event of primary interest; CI, confidence interval; T-DM1, trastuzumab emtansine.

a Grade 1 pneumonitis events occurred in five (0.2%) and three (1.7%) patients in cohorts 1 and 2, respectively; grade 2 pneumonitis events occurred in eight (0.4%) and one (0.6%) patient in cohorts 1 and 2, respectively.

Overall, 93.0% of patients in cohort 1 and 96.1% of patients in cohort 2 reported any treatment-emergent AE (Supplementary Table S1, available at https://doi.org/10.1016/j.esmoop.2022.100561). The majority of treatment-emergent AEs in ≥10% of patients—including nausea, fatigue, and asthenia—were reported more frequently in cohort 1 than in cohort 2, while thrombocytopenia, pyrexia, and cough were reported more frequently in cohort 2. Serious AEs were reported in 21.3% of patients in cohort 1 and 19.9% of patients in cohort 2. Rates of grade ≥3 diarrhea and nausea were low in both cohorts.

In both cohorts, most grade ≥3 thrombocytopenia events were not associated with grade ≥3 hemorrhagic events. Within cohort 1, of 74 patients (3.7%) with grade 3/4 thrombocytopenia, 38 (51.4%) did not experience hemorrhage, 22 (29.7%) experienced grade 1/2 hemorrhage, and 6 (8.1%) experienced grade ≥3 hemorrhage. Within cohort 2, 66 patients (36.5%) had grade 3/4 thrombocytopenia; of these, 39 (59.1%) did not experience hemorrhage, 19 (28.8%) experienced grade 1/2 hemorrhage, and 2 (3.0%) experienced grade 3 hemorrhage (Supplementary Table S2, available at https://doi.org/10.1016/j.esmoop.2022.100561). In cohort 2, the majority of grade ≥3 thrombocytopenia events (128/138) fully resolved, with a duration of ≤15 days for 98/138 of these events (Supplementary Table S3, available at https://doi.org/10.1016/j.esmoop.2022.100561). Of the grade ≥3 thrombocytopenia events that did not fully resolve, one resolved with sequelae, three had an unknown outcome, and six are ongoing.

### Efficacy

**Figure 2:**
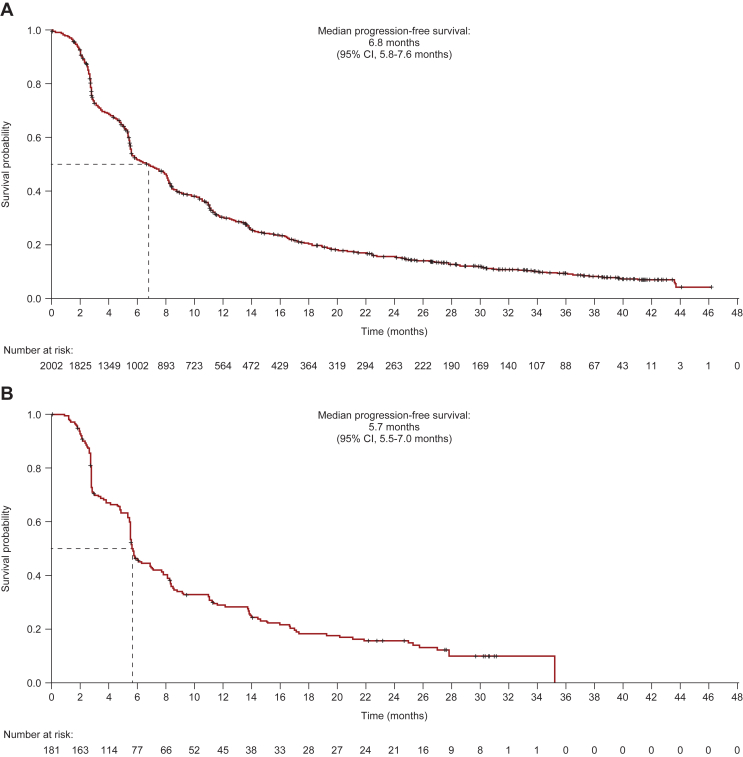
Progression-free survival. (A) Cohort 1—global; (B) cohort 2—Asia. +Censored. Intent-to-treat population (cohort 1, *n* = 2003; cohort 2, *n* = 182). CI, confidence interval.

By the data cut-off, median PFS was 6.8 months (95% CI 5.8-7.6 months) in cohort 1 and 5.7 months (95% CI 5.5-7.0 months) in cohort 2 (Figure 2). In cohorts 1 and 2, respectively, 1730 patients (86.4%) and 147 patients (80.8%) had a PFS event; 273 patients (13.6%) in cohort 1 and 35 patients (19.2%) in cohort 2 were censored.

**Figure 3:**
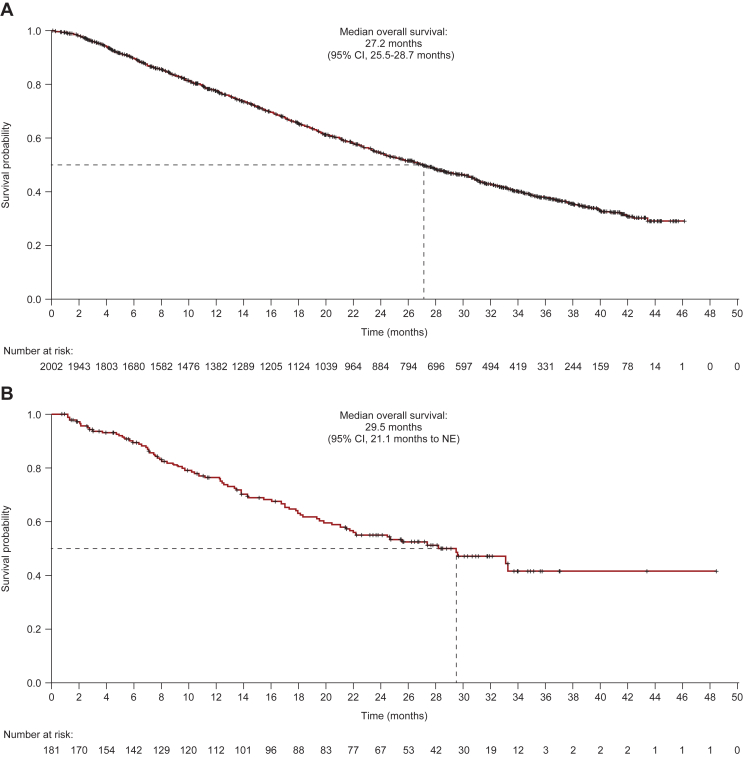
Overall survival. (A) Cohort 1—global; (B) cohort 2—Asia. +Censored. Intent-to-treat population (cohort 1, *n* = 2003; cohort 2, *n* = 182). CI, confidence interval, NE, non-estimable.

In the OS analysis, 1072 patients (53.5%) in cohort 1 and 77 patients (42.3%) in cohort 2 had died. Median OS was 27.2 months (95% CI 25.5-28.7 months) in cohort 1 and 29.5 months (95% CI 21.1 months to non-estimable) in cohort 2; 931 patients (46.5%) in cohort 1 and 105 patients (57.7%) in cohort 2 were censored (Figure 3). In both cohorts, median PFS and median OS decreased with increasing lines of prior therapy (Supplementary Table S4 and Supplementary Figures S1 and S2, available at https://doi.org/10.1016/j.esmoop.2022.100561).

Among 1613 patients in cohort 1 with measurable disease at baseline, the overall response rate was 29.3% (95% CI 27.1% to 31.6%), with 73 (15.4%) and 400 (84.6%) patients, respectively, achieving a complete or partial response. In cohort 2, among 169 patients with measurable disease at baseline, the overall response rate was 29.6% (95% CI 22.8% to 37.1%), with five (10.0%) and 45 (90.0%) patients, respectively, achieving a complete or partial response. Thirty-nine patients from cohort 1 and eight patients from cohort 2 were rolled over to the post-trial access to extension study.

## Discussion

To date, KAMILLA is the largest safety study of patients treated with T-DM1, both globally and in an Asian population. Our analysis of KAMILLA cohorts 1 and 2 provided further evidence that T-DM1 has a manageable safety profile, with no new safety signals reported. These results are in line with prior observations for both global and Asian populations.^4^^,^^5^^,^^12^^,^^14^ PFS and OS results were similar in both cohorts and were consistent with previous T-DM1 studies,^4^^,^^5^ with numerical decreases observed as the number of prior treatment lines increased. Regulatory safety commitments to the EMA were met by this study.

In our analysis, cohort 2 had a higher overall rate of AEPIs than cohort 1 (51.4% versus 23.1%), largely driven by a higher incidence of grade ≥3 thrombocytopenia in cohort 2. The majority of grade ≥3 thrombocytopenia events in cohort 2, however, resolved fully within 15 days. In addition, such events were not associated with a higher risk of grade ≥3 hemorrhage events, similar to what was observed in the EMILIA study.^4^ In EMILIA, the majority of patients with grade ≥3 thrombocytopenia were able to continue T-DM1 treatment with dose modifications.^4^

Several other studies have shown a higher incidence of thrombocytopenia in Asian patients with HER2-positive BC treated with T-DM1. A pooled analysis of single-agent T-DM1 studies in HER2-positive BC reported a higher incidence of grade 3/4 platelet count decrease in Asian versus non-Asian patients (44.4% versus 10.6%).^12^ When data from the T-DM1 clinical trials EMILIA, TH3RESA, and MARIANNE were pooled, Asian patients with a pretreatment platelet count of 100-220 × 10^9^/l had the highest risk of grade ≥3 thrombocytopenia out of six defined subgroups.^15^ Given that Fc-gamma receptors likely play a role in T-DM1-mediated thrombocytopenia, specific Fc polymorphisms that occur more frequently in Asian patients might account for a higher incidence of thrombocytopenia in this population.^12^ T-DM1-induced thrombocytopenia can generally be managed by dose modification, and current guidelines recommend treatment interruption in cases of grade 3 thrombocytopenia and treatment interruption followed by dose reduction for grade 4 thrombocytopenia.^1^

Rates of other AEPIs, including all-grade pneumonitis, as well as rates of grade ≥3 diarrhea and nausea, were low in both cohorts. Pneumonitis, also referred to as interstitial lung disease, is a well-known AE associated with several anti-HER2 agents.^16^, ^17^, ^18^, ^19^ In our analysis, pneumonitis occurred in 1.0% of patients in cohort 1 and 2.2% of patients in cohort 2.

Including these results from KAMILLA cohorts 1 and 2, T-DM1 has consistently demonstrated robust efficacy and a manageable safety profile across trials of advanced BC, irrespective of differences in the study populations, such as age, number of prior treatment lines, and proportion of patients with CNS metastases.^4^^,^^5^^,^^12^^,^^20^ Use of T-DM1 as second-line treatment in patients with HER2-positive BC is further supported by results from real-world studies (Supplementary Table S5, available at https://doi.org/10.1016/j.esmoop.2022.100561).^7^, ^8^, ^9^, ^10^ Although the retrospective nature of some real-world studies may limit availability of safety data, findings from these studies have generally been comparable to those reported in clinical trials.

The treatment landscape for HER2-positive mBC is becoming increasingly complex. Dual blockade with pertuzumab and trastuzumab in combination with chemotherapy remains the preferred first-line regimen for treatment of locally unresectable BC or mBC.^21^, ^22^, ^23^ However, a number of HER2-targeted therapies for advanced HER2-positive BC—including monoclonal antibodies, receptor tyrosine kinase inhibitors, and antibody–drug conjugates alone or in combination—have been approved or are being assessed in clinical trials (Supplementary material, available at https://doi.org/10.1016/j.esmoop.2022.100561).^24^, ^25^, ^26^, ^27^, ^28^, ^29^, ^30^, ^31^ Although the individual indications vary, both T-DM1 and the antibody–drug conjugate trastuzumab deruxtecan (TDXd)^17^^,^^32^ might be considered as treatment options for women with HER2-positive mBC who have previously received two or more anti-HER2-based regimens in the metastatic setting. In a recent head-to-head, phase III trial (DESTINY-Breast03), TDXd treatment resulted in a statistically significant improvement in median PFS compared with T-DM1 and had manageable toxicity in patients with HER2-positive, unresectable BC.^19^ However, increased rates of interstitial lung disease have been observed with TDXd in both the phase II trial that formed the basis for its approval (13.5% of patients receiving TDXd)^17^ and in the phase III study comparing TDXd (10.5%) with T-DM1 (1.9%).^19^ These trial results illustrate the challenges clinicians face in evaluating the risk–benefit balance within an increasingly complex treatment landscape.

This analysis of KAMILLA cohorts 1 and 2 had several limitations. This study was not randomized, and there was no control arm. Additionally, there were some imbalances between cohorts in patient baseline characteristics—such as age, hormone receptor-negative disease status, ECOG performance status, and prior chemotherapy—which may have impacted treatment tolerance.

### Conclusions

Safety and efficacy results from this analysis of global and Asia cohorts from KAMILLA were similar to prior observations. Although the Asia cohort had a higher grade ≥3 thrombocytopenia rate, the majority of these events fully resolved and were not associated with grade 3 hemorrhage. AEs of special interest, such as thrombocytopenia, can generally be managed by dose modifications. The pneumonitis event rate was low. A number of real-world studies have shown a similar benefit–risk balance of T-DM1, reinforcing findings from clinical trials; furthermore, the use of T-DM1 in the second-line setting is supported by clinical guidelines.^21^, ^22^, ^23^ Data from both cohorts reinforce the positive risk–benefit balance of T-DM1 in HER2-positive mBC, further supporting the use of this agent in patients with previously treated disease.

## Funding

This work was supported by 10.13039/100007013F. Hoffmann-La Roche, Switzerland (no grant number). F. Hoffmann-La Roche participated in the study design; in the collection, analysis, and interpretation of data; in the writing of the report; and in the decision to submit the article for publication.

## Disclosure

**RW** reports honoraria, consulting/advisory fees, speaker bureau fees, and travel accommodations from Agendia, Amgen, Aristo, AstraZeneca, Boehringer Ingelheim, Carl Zeiss, Celgene, Clinsol, Daiichi Sankyo, Eisai, Exact Sciences, Genomic Health, GlaxoSmithKline, Hexal, Lilly, Medstrom Medical, Merck Sharp & Dohme (MSD), Mundipharma, NanoString, Novartis, Odonate, Paxman, Palleos, Pfizer, Pierre Fabre, Puma Biotechnology, Riemser, Roche, Sandoz/Hexal, Seagen, Tesaro Bio, Teva, Veracyte, and Viatris. **FM** reports honoraria from Roche; consulting/advisory fees from Novartis, Seagen, Eli Lilly, Pierre Fabre, Roche, Daiichi Sankyo, MSD; and speaker bureau fees from AstraZeneca and Pfizer. **AAT** reports consulting/advisory fees from Bayer, Lilly, and Gilead, and expert testimony fees from Pfizer. **SD** reports consulting/advisory fees paid to her institution from AstraZeneca and Pierre Fabre; research funding paid to her institution from AstraZeneca, Pfizer, Roche/Genentech, Puma Biotechnology, Lilly, Novartis, Sanofi, and Exact Sciences; and travel accommodations from Pfizer, AstraZeneca, and Roche. **HLiu** reports employment at Genentech, Inc., stock ownership at Roche/Genentech, and travel accommodations from Roche/Genentech. **SF** reports employment at Roche. **CPM** reports employment and stock ownership at Roche. **CB** reports stock ownership at Biomarker, MEDSIR, and Tummi; honoraria from Novartis, Roche/Genentech, Pfizer, GlaxoSmithKline, Sanofi, Boehringer Ingelheim, Eisai, MSD, Lilly, Bayer, AstraZeneca, Zodiac Pharma; consulting/advisory fees from Boehringer Ingelheim, Roche/Genentech, Novartis, GlaxoSmithKline, Eisai, Pfizer, AstraZeneca, Libbs, MSD Oncology, United Medical, Lilly; speaker bureau fees paid to his institution from Pfizer, Novartis, Amgen, AstraZeneca, Boehringer Ingelheim, GlaxoSmithKline, Roche/Genentech, Lilly, Sanofi, Taiho Pharmaceutical, Mylan, Merrimack, Merck, AbbVie, Astellas Pharma, BioMarin, Bristol Myers Squibb, Daiichi Sankyo, Abraxis BioScience, AB Science, Asana Biosciences, Medivation, Exelixis, ImClone Systems, LEO Pharma, Millennium, Janssen, Clinica Atlantis, INC Research, Halozyme, Covance, Celgene, inVentiv Health, Merck KGaA, Shanghai Henlius Biotech, Polyphor, and PharmaMar; and travel accommodations from Roche/Genentech, Novartis, Pfizer, BMS Brazil, AstraZeneca, MSD Oncology, and Lilly. All other authors have declared no conflicts of interest.

## Supplementary data

Supplementary Figure S1AB
