# Supplementary material for: Final results of the global and Asia cohorts of KAMILLA, a phase IIIB safety trial of trastuzumab emtansine in patients with HER2-positive advanced breast cancer
Source: ESMO Open. 2022 Sep 7;7(5):100561. doi: 10.1016/j.esmoop.2022.100561 (PMC9588895; doi:10.1016/j.esmoop.2022.100561)
Supplement: Supplementary Figure S1AB [file mmc1.pdf]

Supplemental Appendix to Wuerstlein R, et al. Final results of the global and Asia cohorts of KAMILLA, a phase IIIB safety trial of trastuzumab emtansine in patients with HER2-positive advanced breast cancer

TABLES

Table A.1. Summary of adverse events (N=2183)

Preferred term, n (%)	**Cohort 1 n=2002**		**Cohort 2 n=181**		**Total N=2183**	
	**All grades**	**Grade ≥3**	**All grades**	**Grade ≥3**	**All grades**	**Grade ≥3**
Any TEAE	1862 (93.0)	816 (40.8)	174 (96.1)	123 (68.0)	2036 (93.3)	939 (43.0)
Adverse event individual MedDRA preferred terms (≥10% of patients)						
Nausea	651 (32.5)	14 (0.7)	29 (16.0)	1 (0.6)	680 (31.1)	15 (0.7)
Fatigue	557 (27.8)	50 (2.5)	12 (6.6)	0	569 (26.1)	50 (2.3)
Asthenia	493 (24.6)	35 (1.7)	18 (9.9)	0	511 (23.4)	35 (1.6)
Headache	455 (22.7)	22 (1.1)	19 (10.5)	0	474 (21.7)	22 (1.0)
Epistaxis	405 (20.2)	5 (0.2)	30 (16.6)	0	435 (19.9)	5 (0.2)
Constipation	397 (19.8)	11 (0.5)	10 (5.5)	1 (0.6)	407 (18.6)	12 (0.5)
Pyrexia	347 (17.3)	7 (0.3)	47 (26.0)	0	394 (18.0)	7 (0.3)
Decreased appetite	321 (16.0)	13 (0.6)	16 (8.8)	1 (0.6)	337 (15.4)	14 (0.6)
Vomiting	305 (15.2)	28 (1.4)	18 (9.9)	1 (0.6)	323 (14.8)	29 (1.3)
Dry mouth	283 (14.1)	2 (0.1)	7 (3.9)	0	290 (13.3)	2 (0.1)
Arthralgia	266 (13.3)	3 (0.1)	6 (3.3)	0	272 (12.5)	3 (0.1)
Diarrhea	254 (12.7)	16 (0.8)	9 (5.0)	0	263 (12.0)	16 (0.7)
Cough	220 (11.0)	2 (0.1)	23 (12.7)	0	243 (11.1)	2 (0.1)
Thrombocytopenia	175 (8.7)	55 (2.7)	49 (27.1)	37 (20.4)	224 (10.3)	92 (4.2)
Dyspnea	216 (10.8)	34 (1.7)	2 (1.1)	1 (0.6)	218 (10.0)	35 (1.6)

MedDRA, Medical Dictionary for Regulatory Activities; NA, not available; TEAE, treatment-emergent adverse event.

Table A.2. Cross-tabulation of grade 3/4 platelet count decrease and hemorrhage

		Platelet count decrease based on laboratory data[a]			
		None[b] n (%)	Grade 3 n (%)	Grade 4 n (%)	Total n (%)
Hemorrhage[c]	Cohort 1 (n=2002)[d]				
	No AE	1397 (69.8)[e]	26 (1.3)	12 (0.6)	1435 (71.7)
	Grade 1	390 (19.5)	14 (0.7)	3 (0.1)	407 (20.3)
	Grade 2	105 (5.2)	4 (0.2)	1 (<0.1)	110 (5.5)
	Grade 3	33 (1.6)	5 (0.2)	0	38 (1.9)
	Grade 4	6 (0.3)	0	0	6 (0.3)
	Grade 5	1 (<0.1)	1 (<0.1)	0	2 (0.1)
	Total	1936 (96.7)	50 (2.5)	16 (0.8)	2002 (100)
	Cohort 2 (n=181)				
	No AE	92 (50.8)	28 (15.5)	11 (6.1)	131 (72.4)
	Grade 1	22 (12.2)	10 (5.5)	3 (1.7)	35 (19.3)
	Grade 2	6 (3.3)	3 (1.7)	3 (1.7)	12 (6.6)
	Grade 3	0	0	2 (1.1)	2 (1.1)
	Grade 4	1 (0.6)	0	0	1 (0.6)
	Grade 5	0	0	0	0
	Total	121 (66.9)	41 (22.7)	19 (10.5)	181 (100)

[a]There were no cases of grade 5 platelet count decrease in either cohort.

[b]None included grade 0, 1, or 2 platelet decrease.

[c]Patients with hemorrhage are those with a hemorrhage adverse event of interest. This composite term included preferred terms such as gastric hemorrhage, melena, GI hemorrhage, upper GI hemorrhage, gingival bleeding, epistaxis, intracranial hemorrhage, cerebral hemorrhage, disseminated intravascular coagulation, hematuria, hematoma (not all are listed; list includes those that occurred in ≥2 patients).

[d]Data for Cohort 1 previously published in Montemurro et al, *Eur J Cancer.* 2019;109:92–102.

[e]Four patients were missing data on hemorrhage.

AE, adverse event; GI, gastrointestinal.

Table A.3. Duration of overall grade 3/4 thrombocytopenia events[a]

Duration	Cohort 1[b] (n=2002) No. events = 88	Cohort 2 (n=181) No. events = 138	Total (N=2183) No. events = 226
1-5 days	2 (2.3)	12 (8.7)	14 (6.2)
6-10 days	17 (19.3)	54 (39.1)	71 (31.4)
11-15 days	7 (8.0)	32 (23.2)	39 (17.3)
16-20 days	11 (12.5)	13 (9.4)	24 (10.6)
21-25 days	5 (5.7)	2 (1.4)	7 (3.1)
26-30 days	2 (2.3)	0	2 (0.9)
≥31 days	10 (11.4)	16 (11.6)	26 (11.5)
Unknown[c]	34 (38.6)	9 (6.5)	43 (19.0)

[a]Percentages are based on the total number of events in each group over the course of the study.

[b]Data for Cohort 1 previously published in Montemurro et al, *Eur J Cancer.* 2019;109:92–102.

[c]Not all events have a stop date; therefore, the unknown category includes events whose outcomes are ongoing, unknown, and without end date recorded.

Table A.4. PFS and OS by previous lines of treatment

Number of previous lines of treatment	N	Median PFS (95% CI)	Median OS (95% CI)
**Cohort 1**			
0-1	594	8.3 (7.9-9.2)	31.3 (28.3-34.8)
2	446	6.5 (5.6-8.0)	29.1 (25.5-31.2)
3	358	5.8 (5.5-8.0)	24.1 (21.0-27.4)
4+	517	5.5 (5.3-5.7)	22.5 (20.1-24.4)
Missing	87	8.6 (5.6-13.5)	NE (26.3-NE)
**Cohort 2**			
0-1	62	6.3 (4.6-9.2)	29.6 (21.1-NE)
2	50	5.6 (5.4-7.1)	25.6 (17.0-NE)
3	28	8.5 (5.5-24.9)	NE (10.7-NE)
4+	33	4.8 (2.8-5.7)	18.3 (11.2-NE)
Missing	8	9.9 (2.8-NE)	NE (8.9-NE)
**Total**			
0-1	656	8.2 (7.7-8.6)	31.3 (28.5-34.5)
2	496	6.2 (5.6-7.7)	28.8 (24.9-31.2)
3	386	6.0 (5.5-8.0)	24.5 (21.2-27.9)
4+	550	5.5 (5.3-5.7)	22.0 (19.9-24.1)
Missing	95	8.6 (5.7-13.1)	NE (26.3-NE)

CI, confidence interval; NE, non-estimable; OS, overall survival; PFS, progression-free survival.

Table A.5. Results from real-world studies of T-DM1 in HER2-positive breast cancer

Study	Study design	Key efficacy results	Key safety results
Vic P, et al. 2017[1]	Retrospective, multicenter, observational study	• Of 245 patients treated with T-DM1 and evaluable for efficacy assessment, median PFS and median OS were 6 and 20 months, respectively<br>• No differences in efficacy based on prior pertuzumab treatment	Mild and transient cardiac dysfunction was observed in four (1.6%) patients
Conte B, et al. 2020[2]	Retrospective/prospective multicenter study	• 77 patients with HER2-positive mBC who were treated with T-DM1 after front-line treatment with a taxane plus trastuzumab and pertuzumab were eligible for analysis<br>• Median PFS was 6.3 months<br>• Overall response rate was 27.1%<br>• At data cut-off, the median OS was not reached, and the 1-year OS was 82%.	Safety was not investigated in this study
Bahçeci A, et al. 2021[3]	Retrospective analysis	• Analysis of 414 patients with HER2-positive mBC showed that T-DM1 was more effective in earlier lines of treatment<br>• Median PFS decreased from 37 to 8 months from first to fifth-line therapy<br>• Median OS decreasing from 43 to 17 months from first to fifth-line therapy	The most common grade 3/4 adverse effects were thrombocytopenia (2.7%) and increased serum gamma-glutamyl transferase (2%)
Hardy-Werbin M, et al. 2019[4]	Retrospective analysis	• 15 patients with HER2-positive BC treated with T-DM1<br>• Median PFS was 10 months<br>• Median OS was 34 months	The most common grade 3/4 AEs included elevated transaminases and thrombocytopenia (each 6.7%)

AEs, adverse events; BC, breast cancer; HER2, human epidermal growth factor receptor; OS, overall survival; PFS, progression-free survival; T-DMI, trastuzumab emtansine.

References

1. Vici P, Pizzuti L, Michelotti A, et al. A retrospective multicentric observational study of trastuzumab emtansine in HER2 positive metastatic breast cancer: a real-world experience. *Oncotarget.* 2017;8:56921–56931.
2. Conte B, Fabi A, Poggio F, et al. T-DM1 efficacy in patients with HER2-positive metastatic breast cancer progressing after a taxane plus pertuzumab and trastuzumab: an Italian multicenter observational study. *Clin Breast Cancer.* 2020;20:e181–e187.
3. Bahçeci A, Paydaş S, Ak N, et al. Efficacy and safety of trastuzumab emtansine in HER2 positive metastatic breast cancer: real-world experience. *Cancer Invest.* 2021;39:473–481.
4. Hardy-Werbin M, Quiroga V, Cirauqui B, et al. Real-world data on T-DM1 efficacy – results of a single-center retrospective study of HER2-positive breast cancer patients. *Sci Rep.* 2019;9:12760.

FIGURES

Fig. A.1. PFS by previous lines of treatment

A. Cohort 1 – Global

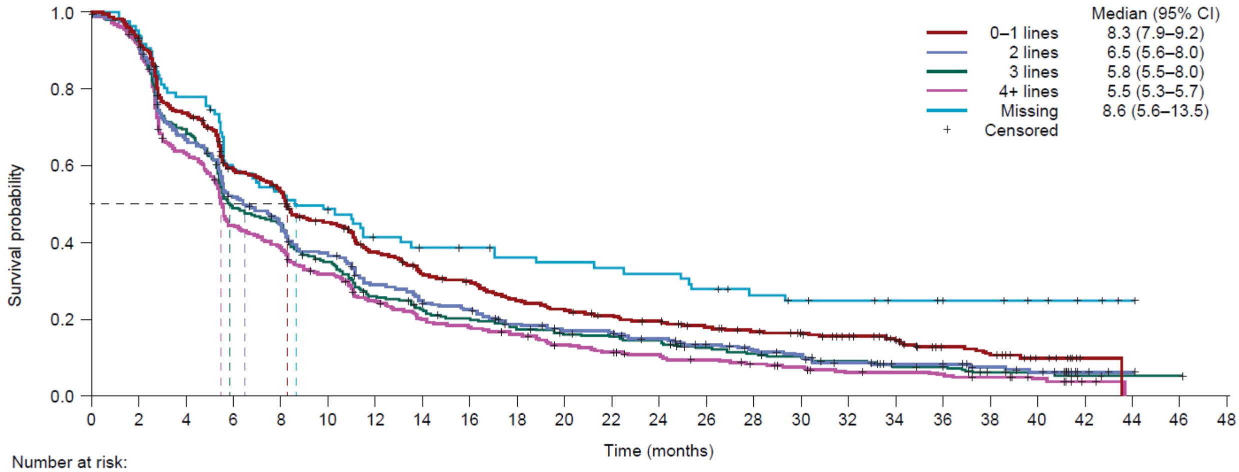

Number at risk:

594	547	432	338	305	252	206	172	159	134	120	109	100	87	76	69	62	43	29	25	12	1	0	0	0
446	403	287	219	191	153	118	100	91	72	63	61	51	42	35	31	22	17	17	12	9	3	1	0	0
358	323	243	172	156	119	87	74	64	56	50	47	43	33	29	26	21	17	16	9	8	2	1	1	0
517	469	320	223	196	159	120	96	86	76	61	53	46	40	33	28	21	19	17	13	8	2	0	0	0
87	83	67	50	45	40	33	30	29	26	25	24	23	20	17	15	14	11	9	8	6	3	1	0	0

B. Cohort 2 – Asia

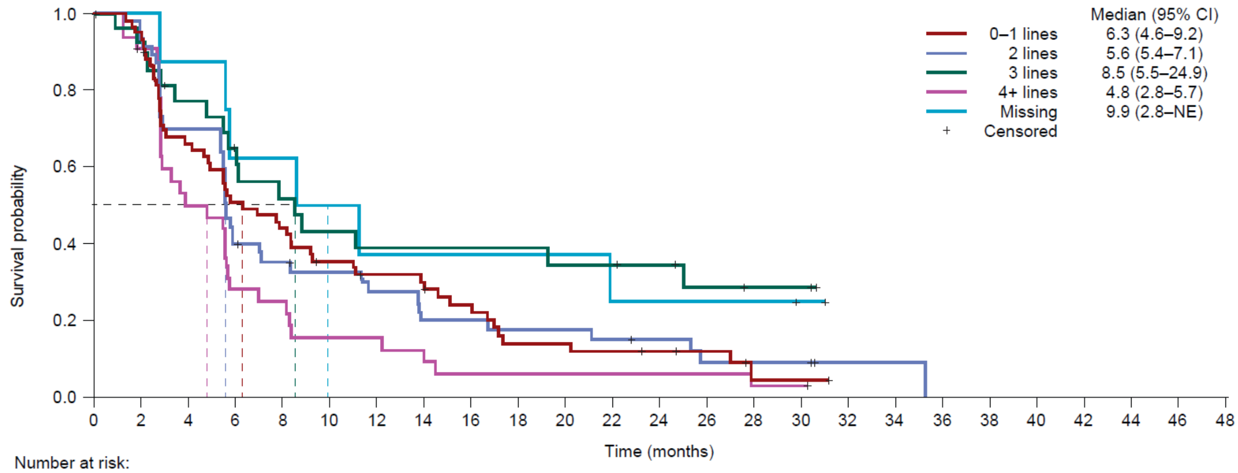

Number at risk:

62	57	39	30	26	20	17	15	11	7	7	6	5	4	1	1	0	0	0	0	0	0	0	0	0
50	44	33	18	15	13	11	8	8	7	7	6	5	3	3	3	1	1	0	0	0	0	0	0	0
28	25	19	15	12	10	9	9	9	9	8	8	7	5	2	2	0	0	0	0	0	0	0	0	0
33	29	16	9	8	5	5	3	2	2	2	2	2	2	1	1	0	0	0	0	0	0	0	0	0
8	8	7	5	5	4	3	3	3	3	3	2	2	2	2	1	0	0	0	0	0	0	0	0	0

+ Censored.

Intent-to-treat population (Cohort 1, n=2003; Cohort 2, n=182).

CI, confidence interval; NE, non-estimable; PFS, progression-free survival.

Fig. A.2. OS by previous lines of treatment

A. Cohort 1 – Global

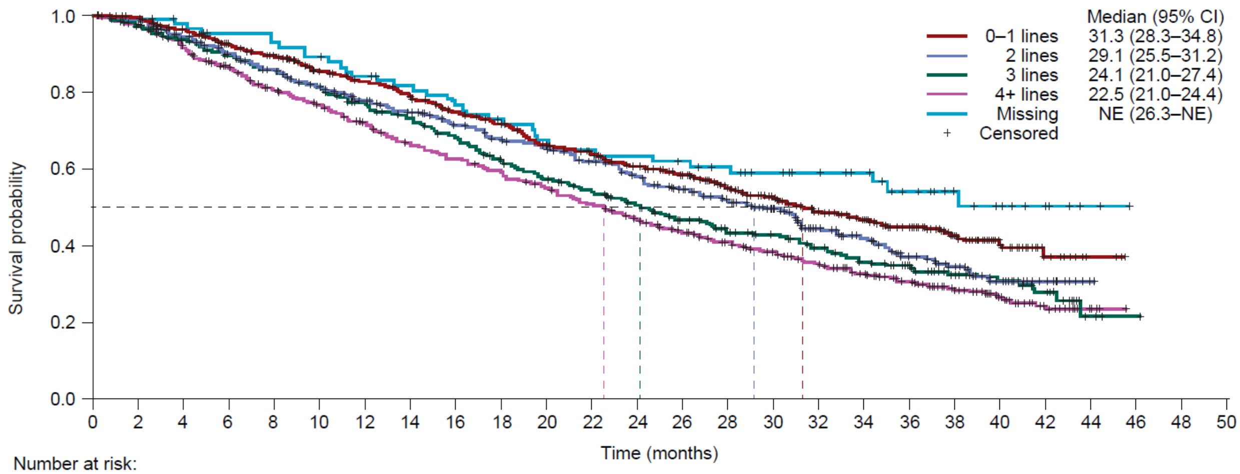

Number at risk:

	0	2	4	6	8	10	12	14	16	18	20	22	24	26	28	30	32	34	36	38	40	42	44	46	48	50
0–1 lines	594	583	557	522	499	462	440	414	387	366	334	318	293	266	231	192	160	135	100	68	40	14	3	0	0	0
2 lines	446	435	397	371	347	326	304	284	365	247	236	218	200	179	152	126	92	75	58	45	27	15	1	0	0	0
3 lines	358	342	316	296	280	259	240	226	208	187	172	157	143	124	109	102	90	76	65	47	34	17	4	1	0	0
4+ lines	517	497	451	413	380	356	330	301	285	268	246	226	204	183	165	144	123	107	89	70	47	26	4	0	0	0
Missing	87	86	82	78	76	73	68	64	60	56	51	45	44	42	39	33	29	26	19	14	11	6	2	0	0	0

B. Cohort 2 – Asia

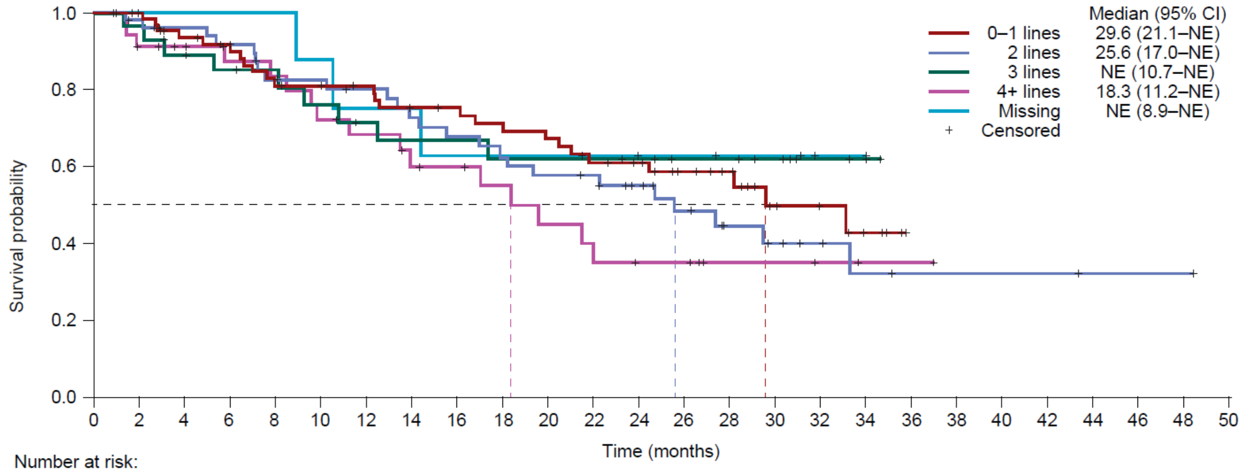

Number at risk:

	0	2	4	6	8	10	12	14	16	18	20	22	24	26	28	30	32	34	36	38	40	42	44	46	48	50
0–1 lines	62	60	54	50	45	43	42	38	37	34	33	29	27	19	16	9	7	4	0	0	0	0	0	0	0	0
2 lines	50	47	44	40	35	34	32	29	27	25	23	22	18	14	10	8	6	4	2	2	2	2	1	1	1	0
3 lines	28	26	22	21	19	17	15	14	14	13	13	13	12	10	10	7	3	2	0	0	0	0	0	0	0	0
4+ lines	33	29	26	23	22	19	17	14	13	11	9	8	6	6	3	3	2	1	1	0	0	0	0	0	0	0
Missing	8	8	8	8	8	7	6	6	5	5	5	5	4	4	3	3	1	1	0	0	0	0	0	0	0	0

+ Censored.

Intent-to-treat population (Cohort 1, n=2003; Cohort 2, n=182).

CI, confidence interval; NE, non-estimable; OS, overall survival.

SUPPLEMENTAL DISCUSSION

Overview of HER2-targeted therapies for advanced HER2-positive breast cancer

In addition to trastuzumab emtansine (T-DM1), a number of human epidermal growth factor receptor 2 (HER2)–targeted therapies for advanced HER2-positive breast cancer (BC)—including monoclonal antibodies, receptor tyrosine kinase inhibitors, and antibody-drug conjugates alone or in combination—have been approved or are undergoing clinical trials in the advanced/metastatic setting. The dual blockade with pertuzumab and trastuzumab in combination with chemotherapy remains the preferred first-line regimen for treatment of locally unresectable BC or metastatic BC (mBC).[1-3] Following the approval of lapatinib for treatment of HER2-positive mBC,[4] two other tyrosine kinase inhibitors – neratinib and tucatinib – were approved for HER2-positive mBC in the United States.[5-8] Neratinib was approved for the treatment of patients with HER2-positive advanced BC or mBC who previously received ≥2 anti-HER2 regimens in the metastatic setting, based on results from the phase III NALA trial.[5,7] In NALA, the most common TEAEs were diarrhea, nausea, palmar-plantar erythrodysesthesia syndrome, and vomiting; the rate of grade 3 diarrhea was 24.4% in the neratinib arm and 12.5% in the lapatinib comparator arm.[7] Following results from the phase III HER2CLIMB study, tucatinib in combination with trastuzumab and capecitabine was approved for the treatment of patients with HER2-positive advanced unresectable BC or mBC who received ≥1 prior anti-HER2 regimens in the metastatic setting.[6,8] In HER2CLIMB, the most common adverse events in the tucatinib-combination group were diarrhea, palmar-plantar erythrodysesthesia syndrome, nausea, fatigue, and vomiting; grade ≥3 diarrhea and elevated aminotransferase levels were higher in the tucatinib-combination group than in the placebo-combination group.[8] A phase Ib trial in heavily pretreated patients with HER2-positive mBC demonstrated that tucatinib in

combination with T-DM1 had acceptable toxicity, with no drug-drug interactions; grade ≥3 treatment-related adverse events included thrombocytopenia and hepatic transaminitis.[9] Pyrotinib has been approved conditionally in China in combination with capecitabine for the treatment of patients with HER2-positive advanced BC or mBC who have previously been treated with anthracycline or taxane chemotherapy.[10] This conditional approval was based on a phase II study that demonstrated statistically significant better overall response rate and PFS with pyrotinib plus capecitabine versus lapatinib plus capecitabine; the most frequent grade 3–4 adverse events were hand-foot syndrome (24.6% versus 20.6%), diarrhea (15.4% versus 4.8%), and decreased neutrophil count (9.2% versus 3.2%).[10,11]

Overview of ongoing trials investigating combination therapies for advanced HER2-positive breast cancer

A number of ongoing trials are currently investigating combination therapies. The randomized, double-blind, placebo-controlled phase III HER2CLIMB-02 trial is currently evaluating the efficacy and safety of tucatinib plus T-DM1 in patients with unresectable, HER2-positive locally advanced BC or mBC.[12] A phase III study of T-DM1 in combination with atezolizumab or placebo for patients with HER2-positive and programmed death ligand–positive locally advanced BC or mBC (KATE3) is currently recruiting.[13] Trastuzumab deruxtecan in combination with pertuzumab is under evaluation in the phase 3 DESTINY-Breast09 study (NCT04784715).The randomized, open-label, phase III PATINA trial is investigating palbociclib in combination with anti-HER2 and endocrine therapy for the treatment of hormone receptor positive/HER2-positive mBC (NCT02947685). Alpelisib in combination with trastuzumab and pertuzumab is being evaluated in the phase III EPIK-B2 trial as maintenance therapy for patients with HER2-positive advanced breast cancer with a *PIK3CA* mutation (NCT04208178).

References

1. Referenced with permission from the NCCN Clinical Practice Guidelines in Oncology (NCCN Guidelines®) for Breast Cancer Guidelines V.8.2021. © National Comprehensive Cancer Network, Inc. 2021. All rights reserved. Accessed February 17, 2022. To view the most recent and complete version of the guideline, go online to NCCN.org. NCCN makes no warranties of any kind whatsoever regarding their content, use or application and disclaims any responsibility for their application or use in any way.

2. Cardoso F, Paluch-Shimon S, Senkus E, et al. 5th ESO-ESMO international consensus guidelines for advanced breast cancer (ABC 5). *Ann Oncol.* 2020;31:1623–1649.

3. Thill M, Friedrich M, Kolberg-Liedtke C, et al. AGO recommendations for the diagnosis and treatment of patients with locally advanced and metastatic breast cancer: update 2021. *Breast Care (Basel)*. 2021;16:228–235.

4. Tykerb® prescribing information: Novartis Pharmaceuticals Corporation. https://www.accessdata.fda.gov/drugsatfda_docs/label/2018/022059s023lbl.pdf; 2018. Accessed 16 July 2021.

5. Nerlynx® prescribing information: Puma Biotechology, Inc. https://www.accessdata.fda.gov/drugsatfda_docs/label/2020/208051s005s006lbl.pdf; 2020. Accessed 16 July 2021.

6. Tukysa™ prescribing information: Seattle Genetics, Inc. https://www.accessdata.fda.gov/drugsatfda_docs/label/2020/213411s000lbl.pdf; 2020. Accessed 9 August 2021.

7. Saura C, Oliveira M, Feng YH, et al. Neratinib plus capecitabine versus lapatinib plus capecitabine in HER2-positive metastatic breast cancer previously treated with ≥2 HER2-directed regimens: phase III NALA trial. *J Clin Oncol.* 2020;38:3138–3149.

8. Murthy RK, Loi S, Okines A, et al. Tucatinib, trastuzumab, and caepcitabine for HER2-positive metastatic breast cancer. *N Engl J Med.* 2020;13:597–609.

9. Borges VF, Ferrario C, Aucoin N, et al. Tucatinib combined with ado-trastuzumab emtansine in advanced ERBB2/HER2-positive metastatic breast cancer: a phase 1b clinical trial. *JAMA Oncol.* 2018;4:1214–1220.

10. Blair HA. Pyrotinib: first global approval. *Drugs.* 2018;78:1751–1755.

11. Ma F, Ouyang Q, Li W, et al. Pyrotinib or lapatinib combined with capecitabine in HER2-positive metastatic breast cancer with prior taxanes, anthracyclines, and /or trastuzumab: a randomized/phase II study. *J Clin Oncol* 2019;37:2610–2619.

12. Hurvitz SA, Vahdat LT, Harbeck N. HER2CLIMB-02: a randomized, double-blind, phase III study of tucatinib or placebo with T-DM1 for unresectable locally advanced or metastatic HER2+ breast cancer. *Ann Oncol.* 2020;31(suppl):S390.

13. Loi S, Schneeweiss A, Song E. KATE3: a phase III study of trastuzumab emtansine (T-DM1) in combination with atezolizumab or placebo in patients with previously treated HER2-positive and PD-L1-positive locally advanced or metastatic breast cancer. *Ann Oncol* 2021;32:S457–S515.
